# Gray matter reduction in high-risk subjects, recently diagnosed and chronic patients with schizophrenia: A revised coordinate-based meta-analysis

**DOI:** 10.1192/j.eurpsy.2021.360

**Published:** 2021-08-13

**Authors:** C. Brasso, D. Liloia, F. Cauda, L. Mancuso, A. Nani, J. Manuello, T. Costa, S. Duca, P. Rocca

**Affiliations:** 1 Department Of Neuroscience, University of Turin, Torino, Italy; 2 Department Of Psychology, University of Turin, TORINO, Italy; 3 Neuroradiology, Koelliker Hospital, Torino, Italy

**Keywords:** voxel-based morphometry, behavioral analysis, salience network, psychosis

## Abstract

**Introduction:**

Characterizing neuroanatomical markers of different stages of schizophrenia (SZ) to assess of how the disorder develops is extremely important for the clinical practice. It still remains uncertain how abnormalities are formed as SZ progresses.

**Objectives:**

We reviewed and analyzed 113 voxel based morphometry studies on people at risk of or with schizophrenia to assess GM alterations at different stages of the disorder and to functionally characterize these GM variations.

**Methods:**

We performed a meta-analysis of voxel-based morphometry studies of genetic and clinical high-risk subjects (g-/c-HR), recently diagnosed (RDSZ) and chronic SZ patients (ChSZ). We quantified gray matter (GM) changes associated with these four conditions and compared them with contrast and conjunctional data. We performed the behavioral analysis and networks decomposition of alterations to obtain their functional characterization.

**Results:**

Compared to previous investigations, results reveal a robust cortical-subcortical, left-to-right homotopic progression of GM loss. The right anterior cingulate is the only altered region in all conditions. Contrast analyses show left-lateralized insular, amygdalar and parahippocampal GM reduction in RDSZ, which appears bilateral in ChSZ. An overlap between RDSZ and ChSZ is observed in the left insula, amygdala, precentral and inferior frontal gyri. Functional decomposition shows involvement of the salience network, with an enlargement of the sensorimotor network in RDSZ and the thalamus-basal nuclei network in ChSZ.

**Figure:**
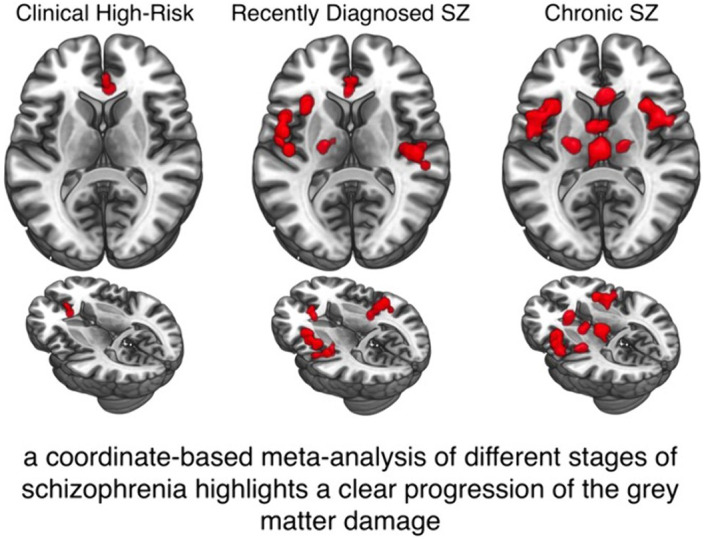

**Conclusions:**

These results can help the research on diagnostic and neuroimaging biomarkers of SZ staging, as well as on the identification of new therapeutics neuroanotomic targets that could be addressed with focused magnetic or non-invasive electric stimulation.

**Disclosure:**

No significant relationships.

